# Impact of Liver Metastases on Outcomes With Chemotherapy or Immune Checkpoint Inhibitors in Pancreatic Cancer

**DOI:** 10.1002/iid3.70545

**Published:** 2026-10-06

**Authors:** Laura K. Kjær, Susann Theile, Kasper Madsen, Julia S. Johansen, Morten Orebo Holmström, Mads Hald Andersen, Inna M. Chen

**Affiliations:** ^1^ Department of Oncology Copenhagen University Hospital ‐ Herlev and Gentofte Herlev Denmark; ^2^ National Center for Cancer Immune Therapy, Department of Oncology Copenhagen University Hospital ‐ Herlev and Gentofte Herlev Denmark; ^3^ Department of Medicine Copenhagen University Hospital ‐ Herlev and Gentofte Herlev Denmark; ^4^ Department of Clinical Medicine, Faculty of Health and Medical Sciences Copenhagen University Copenhagen Denmark

**Keywords:** chemotherapy, immune checkpoint inhibitors, liver metastases, pancreatic cancer, tumor microenvironment

## Abstract

**Background:**

Pancreatic cancer (PC) is on the rise worldwide, with significant improvements in survival rates still awaited. Most patients are diagnosed at an advanced stage, often with liver metastases (LM), associated with poor prognosis and immunosuppression. This study aimed to evaluate the impact of LM on outcomes in patients with advanced PC treated with chemotherapy or immune checkpoint inhibitors (ICIs).

**Material and Methods:**

We performed post‐hoc survival analyses using data from a chemotherapy‐based study (ClinicalTrials.gov ID: NCT02767557) (*n* = 147) and ICI‐based studies (NCT04258150, NCT02866383, and NCT05116917) (*n* = 129). Patients were stratified based on the presence of LM. Exploratory analyses within the LM subgroup were adjusted for absolute lymphocyte counts (ALCs), neutrophile‐lymphocyte‐ratios (NLRs), interleukin (IL)−6, IL‐8, carbohydrate antigen 19‐9, C‐reactive protein, and YKL‐40.

**Results:**

In the chemotherapy cohort, overall survival (OS) was significantly lower in patients with LM (*n* = 107) (7.0 months, 95% confidence interval (CI): 6.0–8.8) compared to those without LM (*n* = 40) (12.0 months, 8.7–18.0; log‐rank *p* = 0.002). Progression‐free survival (PFS) was similarly reduced in the LM group (5.0 months, 3.6–5.7) versus the non‐LM group (7.5 months, 5.4–9.0; *p* = 0.016). In the ICI cohort, no significant differences in OS were observed between patients with (*n* = 111) and without LM (*n* = 18). Adjusted analyses indicated that higher IL‐6 and YKL‐40 were associated with increased mortality risk in patients receiving chemotherapy.

**Discussion:**

LM were associated with significantly shorter OS and PFS in patients with PC receiving chemotherapy. No survival impact of LM was observed in patients treated with ICIs. These findings highlight the need to consider metastatic burden when stratifying treatment outcomes in future studies.

## Summary

Liver metastases (LM) are associated with poor outcomes in patients with pancreatic cancer (PC) treated with chemotherapy but not in those receiving immune checkpoint inhibitors (ICIs). Baseline elevated serum IL‐6 and YKL‐40 are prognostic biomarkers in patients with PC and LM treated with chemotherapy; this association is not observed in patients receiving ICIs. Further studies are needed to clarify the impact of LM in patients with advanced PC receiving ICIs.

**Abbreviations**
ALC: absolute lymphocytes count
CA: carbohydrate antigen
CRP: C‐reactive protein
ICI: immune checkpoint inhibitor
IL: interleukin
LM: liver metastases
LMR: lymphocyte‐to‐monocyte ratio
NLR: neutrophile‐lymphocyte‐ratio
OS: overall survival
PC: pancreatic cancer
PFS: progression‐free survival
PS: performance status
SBRT: stereotactic body radiotherapy
TAMs: tumor‐associated macrophages
TME: tumor microenvironment

## 1 Background

Advanced pancreatic cancer (PC) remains largely resistant to treatment, contributing to its poor prognosis and substantial global health burden as the seventh leading cause of cancer‐related death worldwide [1]. In the United States alone, PC is already the third leading cause of cancer death and is projected to become the second by 2040 [2].

A key challenge in PC management is that most patients are diagnosed at an advanced, unresectable stage, where treatment options are primarily palliative [3]. Eligible chemotherapy regimens provide only modest benefits, with a median overall survival (OS) of less than a year [4, 5]. Despite the transformative success of immune checkpoint inhibitors (ICIs) in multiple malignancies, their efficacy in patients with PC has been disappointing [6].

Liver metastases (LM) are present in nearly all patients with advanced PC [7] and are associated with reduced survival across various cancer types treated with ICIs [8, 9]. This is likely due to the liver's highly immunosuppressive tumor microenvironment (TME), which protects against autoimmunity by tolerating accumulated antigens and toxins. Emerging data suggest distinct liver immune profiles in extrahepatic metastatic PC (upregulation of immune‐related genes suggesting an anti‐metastatic phenotype in the liver) compared with liver‐metastatic PC and patients with no evaluable disease after resection at follow‐up (median follow‐up: 36 months) [10]. Another recent study found that distinct neighborhood‐defined immune profiles in the primary tumor may correlate with metastatic tropism of relapse (liver‐recurrence vs. extrahepatic) [11]. The immunosuppressive TME in PC, compounded with LM, exacerbates both local and systemic immune suppression [12, 13].

Preclinical models suggest that LM‐driven immune dysfunction involves an increased fraction of FasL+CD11b+F4/80+ monocyte‐derived macrophages, which promote Fas+CD8+T‐cell apoptosis in the liver and deplete cytotoxic CD8+T‐cell from systemic circulation [13]. Moreover, PC tumor cells, immune cells, and inflammatory cytokines in the peripheral blood and portal veins further contribute to systemic immunosuppression [14].

Beyond the liver, immune dysregulation in PC involves a shift in the balance of tumor‐infiltrating lymphocytes and macrophages. Particularly, CD4+ and CD8+T cells play key roles in tumor suppression by inducing cytotoxic cell death and inhibiting tumor cell proliferation [15]. Conversely, monocytes differentiate into tumor‐associated macrophages (TAMs), with the TME facilitating immune invasion and metastasis [15]. Elevated macrophages and neutrophil infiltration in the PC TME correlate with escape mechanisms and resistance to immunotherapy [14].

Despite these challenges, emerging therapeutic strategies offer potential. ICIs combined with radiotherapy have shown promise in inducing systemic responses in a subgroup of patients [16]. Preclinical PC models demonstrate that liver‐targeted radiotherapy can restore ICI efficacy by reducing hepatic tumor burden, increasing hepatic T‐cell infiltration, and decreasing immunosuppressive myeloid cells [13]. These findings underscore the radiotherapy's potential to overcome the immunosuppressive barriers imposed by LM in PC.

Systemic immune biomarkers provide additional prognostic insights in PC. In several malignancies, including PC, a low pretreatment absolute lymphocyte count (ALC) has been associated with worse outcomes and diminished responses to ICIs [9, 17]. Similarly, a low pretreatment lymphocyte‐to‐monocyte ratio (LMR) has been linked to poor prognosis in PC and other cancers [15], while a high neutrophil‐to‐lymphocyte ratio (NLR) correlates with shorter survival [14]. These observations indicate that systemic inflammation and immune suppression contribute to poor outcomes in PC and may be influenced by the presence of LM. Additionally, elevated levels of inflammatory markers, such as interleukin (IL)−6, IL‐8, YKL‐40 (also known as chitinase‐3‐like protein 1 (CHI3L1)), C‐reactive protein (CRP), and the widely used tumor marker carbohydrate antigen (CA) 19‐9, have been associated with worse prognosis in PC, consistent with an immunosuppressive systemic milieu that could be further exacerbated by LM [18, 19].

Given this highly immunosuppressive environment of LM, we hypothesized that patients with advanced PC and LM derive less benefit from ICI‐based regimens due to the immune‐modulatory effects of LM, which may impair the systemic immune response and TME. To investigate this, we analyzed pooled data from three ICI‐based studies: Triple‐R, CheckPAC, and Influence [16, 20, 21].

To validate this, we analyzed data from the PACTO study [22], a chemotherapy‐based regimen without ICIs, to determine whether LM also serve as a negative prognostic factor in patients receiving chemotherapy. Additionally, we assessed systemic immune biomarkers to explore their association with the presence of LM and survival outcomes, further contextualizing our findings within the broader framework of LM‐driven immune suppression observed across malignancies.

## 2 Materials and Methods

### 2.1 Patient Population

Patients with advanced PC were included from four previously conducted clinical trials. The PACTO study (ClinicalTrials.gov ID: NCT02767557) [22] represented the chemotherapy regimen group. For the ICI regimen group, data were pooled from three studies: Triple‐R (ClinicalTrials.gov ID: NCT0425815), CheckPAC (ClinicalTrials.gov ID: NCT02866383) and Influence (ClinicalTrials.gov ID: NCT05116917) [16, 20, 21].

The PACTO study was a randomized phase 2 study, which included 147 patients with advanced, treatment‐naïve PC, an Eastern Cooperative Oncology Group performance status (ECOG PS) of 0‐1 and CRP > 10 mg/L. Patients were randomized to receive gemcitabine (1000 mg/m^2^) and nab‐paclitaxel (125 mg/m^2^) with or without tocilizumab, an IL‐6 receptor antibody (8 mg/kg). Details are provided in the original study [22].

The Triple‐R study included 26 patients with advanced refractory PC and ECOG PS 0‐1, who had progressed after at least one line of chemotherapy. Patients received SBRT of 15 Gy × 1 fraction, targeting a feasible metastasis or primary tumor in situ, combined with ipilimumab (1 mg/kg every 6 weeks), nivolumab (3 mg/kg every 2 weeks), and tocilizumab (8 mg/kg every 4 weeks) for up to 1 year or until radiological/clinical progression or unacceptable toxicity occurred. Details are provided in the original study [20].

The CheckPAC study was a randomized phase 2 study, which included 84 patients with advanced, refractory PC and ECOG PS 0‐1, who had received at least one line of chemotherapy regimen and progressed prior to enrollment. Eligible patients received stereotactic body radiotherapy (SBRT) 15 Gy × 1 fraction against a feasible metastasis or primary tumor in situ, followed by either nivolumab (3 mg/kg every 2 weeks) alone or in combination with ipilimumab (1 mg/kg every 6 weeks) for up to 1 year or until radiological/clinical progression or unacceptable adverse events. Details are provided in the original study [16].

The Influence study included 19 patients with refractory, advanced PC and ECOG PS 0‐1, who had progressed after at least one line of chemotherapy. Eligible patients received SBRT 15 Gy × 1 fraction against a feasible metastasis or primary tumor in situ, a single dose of the seasonal influenza vaccine (0.5 mL) combined with ipilimumab (1 mg/kg every 6 weeks, up to 2 doses) and nivolumab (3 mg/kg every 2 weeks) for up to 1 year or until radiological/clinical progression or unacceptable adverse events. Details are provided in the original study [21].

The PACTO study was used as the control group since these patients did not receive ICIs or SBRT. The CheckPAC, Triple‐R, and Influence studies were combined for analyses, as all three included participants who received a combination of SBRT and ICI regimens.

### 2.2 Biomarker Analysis

Leukocytes, neutrophils, lymphocytes, platelets, and hemoglobin were measured in EDTA‐anticoagulated whole blood using a Sysmex XN‐9000 automated hematology analyzer (Sysmex Corporation, Kobe, Japan) at the Department of Clinical Biochemistry, Herlev Hospital. Leukocyte and differential counts were determined by fluorescence flow cytometry, platelet counts by hydrodynamically focused impedance, and hemoglobin by the cyanide‐free sodium lauryl sulfate method [23, 24]. CA19‐9 was measured using the IMMULITE 2000 GI‐MA assay (Siemens, Catalogue Number L2KG12). CRP was measured using a sensitive CRP ultra‐ready‐to‐use liquid assay reagent by an immunoturbidimetric method on a fully automated chemistry analyzer (Kit‐test SENTINEL CRP Ultra (UD), 11508 UD‐2.0/02 2015/09/23).

The serum samples for IL‐6, IL‐8 and YKL‐40 measurements were collected before first treatment in the different studies according to the BIOPAC study (“BIOmarkers in patients with PAncreatic Cancer (BIOPAC) – can they provide new information of the disease and improve diagnosis and prognosis of the patients?” Clinicaltrials.gov NCT03311776) [24]. IL‐6 was measured using a high‐sensitive enzyme‐linked immunosorbent assay (ELISA) (Quantikine HS600B, R&D Systems, Abingdon, UK). IL‐8 was measured using an ELISA (S8000C, R&D Systems, Abingdon, UK). YKL‐40 was measured using an ELISA (Quidel Corporation, San Diego, California, USA).

### 2.3 Statistical Analyses

All analyses are post hoc, as the original studies were not specifically designed to evaluate the impact of LM. For baseline characteristics, the appropriate statistical test was selected based on the distribution of the data: either a non‐parametric Wilcoxon test or a *t*‐test for continuous data, and a chi‐squared test for the categorical data. These analyses were conducted for both the primary and secondary outcomes.

Baseline characteristics are expressed as numbers, frequencies, and medians (25th–75th percentile). CA19‐9, CRP, IL‐6, IL‐8, YKL‐40 and circulating blood cells were stratified according to commonly used cut‐off levels. Two level categories (low: ↓ and high: ↑) were: ≤ 37 and > 37 U/L for CA‐19‐9; ≤ 10 and > 10 mg/L for CRP; ≤ 5 and > 5 ng/L for IL‐6; ≤ 78 and > 78 ng/L for IL‐8; ≤ 200 and > 200 µg/L for YKL‐40; median cut‐off values for ALC was ≤ 5 and > 5 for NLR.

For primary outcomes, Kaplan‐Meier estimates were used to assess time‐to‐event measures. Cox proportional hazard regression analyses were used to estimate hazard ratios (HRs) with 95% confidence intervals (CIs). Both univariate and multivariate models were performed, adjusting for covariates including age, sex, ECOG PS, CRP, CA 19‐9, IL‐6, IL‐8, YKL‐40, ALC and NLR. Biomarker concentrations were log‐transformed (log2) and entered as continuous covariates or as high versus normal levels according to predefined cut‐off levels. A significance level of *p* < 0.05 was used as the threshold for statistical significance. Statistical analyses were conducted using R software (version 4.3.2) [25].

## 3 Results

A total of 276 patients with advanced PC were included in the analysis, as depicted in Figure 1. The chemotherapy group included 107 patients with LM and 40 patients without LM. The ICI group consisted of 111 patients with LM and 18 patients without. Thus, the majority of patients in both treatment groups had LM in each group.

**Figure 1:**
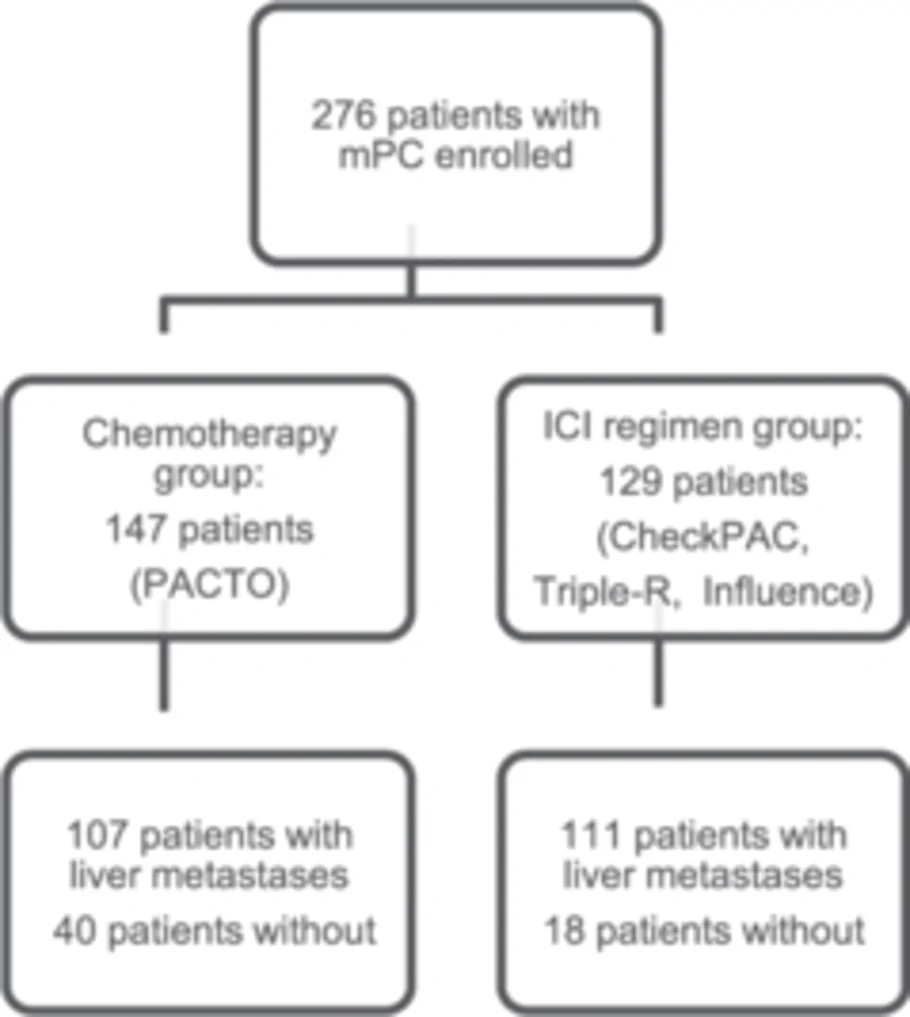
Study flow depicted. ICI, immune checkpoint inhibitor; mPC, metastatic pancreatic cancer.

In the chemotherapy group, relevant baseline characteristics stratified by LM are depicted in Table 1. Key findings indicate that sex, age, and ECOG PS were comparable between groups, while CRP, CA19‐9, IL‐6, IL‐8, and NLR levels were significantly higher in the LM group (Table 1, left). No difference was seen for baseline ALC and YKL‐40. Among chemotherapy‐treated patients, the proportion achieving a PR was numerically higher in patients with LM than in those without LM (39% vs. 25%), although the difference was not statistically significant and did not translate into improved PFS or OS (Figure 2A,B).

**Table 1:** Baseline characteristics of patients in the chemotherapy regimen group and the immune checkpoint inhibitor (ICI) regimen group stratified by liver metastases (LM).

	Chemotherapy regimen group				ICI regimen group			
	*N*	No LM *N* = 40	LM *N* = 107	*p*‐value*	*N*	No LM *N* = 18	LM *N* = 111	*p*‐value*
Sex, *N* (%)	147			0.6	129			0.4
Female		18 (45%)	43 (40%)			10 (56%)	50 (45%)	
Male		22 (55%)	64 (60%)			8 (44%)	61 (55%)	
Age, years^#^	147	70 (60, 73)	66 (59, 73)	0.3	129	65 (55, 69)	64 (55, 70)	0.9
ECOG PS	147			> 0.9	129			0.7
0		15 (38%)	40 (37%)			9 (50%)	60 (54%)	
1		25 (63%)	67 (63%)			9 (50%)	51 (46%)	
CRP, mg/L^#^	146	17 (12, 31)	33 (16, 67)	0.002	129	4 (4, 14)	18 (4, 52)	0.020
CA 19‐9, U/L^#^	147	909 (19, 5655)	3010 (218, 18400)	0.013	128	993 (222, 4840)	4345 (490, 18500)	0.037
IL‐6, ng/L^#^	143	6 (3, 10)	11 (4, 21)	0.004	116	4 (3, 20)	10 (4, 19)	0.2
IL‐8, ng/L^#^	143	33 (26, 53)	81 (41, 200)	< 0.001	116	34 (26, 54)	54 (33, 101)	0.027
YKL‐40, µg/L^#^	143	171 (74, 241)	160 (109, 271)	0.4	116	162 (79, 233)	208 (102, 368)	0.2
ALC, ×10^9^/L^#^	142	1.4 (0.8, 1.9)	1.3 (1.0, 1.8)	0.6	129	1.2 (0.8, 1.7)	1.4 (1.0, 1.8)	0.3
NLR^#^	142	4.0 [2.8–5.8]	5.4 [3.4–8.7]	0.039	129	3.0 (1.6, 4.7)	3.7 (2.8, 5.1)	0.2
BOR, *N* (%)	147			0.12	129			0.6
Complete response		0 (0%)	0 (0%)			2 (11%)	5 (4.5%)	
Partial response		10 (25%)	42 (39%)			2 (11%)	19 (17%)	
Stable disease		19 (48%)	32 (30%)			12 (67%)	69 (62%)	
Progressive disease		11 (28%)	33 (31%)			2 (11%)	18 (16%)	
Prior treatment					129			0.069
1						3 (17%)	47 (42%)	
2						11 (61%)	50 (45%)	
3						2 (11%)	11 (9.9%)	
≥4						2 (11%)	3 (2.7%)	

Note: *Pearson's Chi‐squared test; Wilcoxon rank sum test; Fisher's exact test. ^#^values are median (range).

Continuous variables are presented as median (interquartile range), and categorical variables as *n* (%).

In the chemotherapy group, data were missing for CRP in 1 patient, ALC in 5 patients, and IL‐6, IL‐8, and YKL‐40 in 4 patients each. In the ICI regimen group, data were missing for CA 19‐9 in 1 patient and for IL‐6, IL‐8, and YKL‐40 in 13 patients each.

Abbreviations: ALC, absolute lymphocyte count; CA 19‐9, carbohydrate antigen 19‐9; CRP, C‐reactive protein; IL‐6, interleukin‐6; IL‐8, interleukin‐8; *N*, number; NLR, neutrophil‐to‐lymphocyte ratio; YKL‐40, chitinase 3‐like 1 protein (CHI3L1).

**Figure 2:**
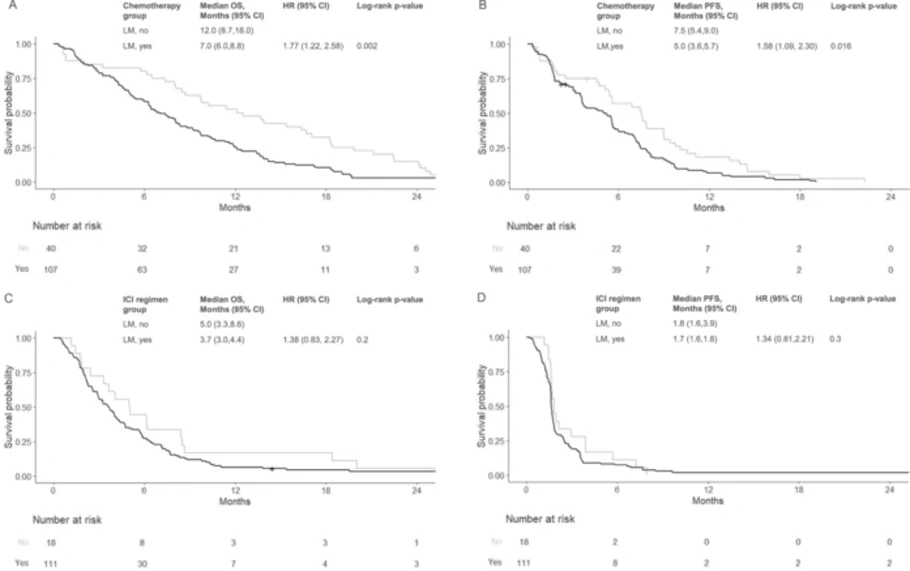
Kaplan–Meier curves for survival according to liver metastasis status. (A) Overall survival in the chemotherapy regimen group. (B) Progression‐free survival in the chemotherapy regimen group. (C) Overall survival in the pooled Triple‐R, CheckPAC, and INFLUENCE ICI regimen group. (D) Progression‐free survival in the pooled Triple‐R, CheckPAC, and INFLUENCE ICI regimen group.

Baseline characteristics for patients treated with ICI regimen group are shown in Table 1 (right). Sex, age, ECOG PS, and number of previous treatment lines (chemotherapy regimens) were comparable between LM group and non‐LM groups. All patients had refractory PC and progressed on first‐line chemotherapy. Like the chemotherapy group, CRP, CA 19‐9, and IL‐8 levels were higher in the LM group. While IL‐6 levels also appeared higher, the difference was not statistically significant. No significant differences were seen in ALC, NLR, or YKL‐40 between the two ICI groups. Best overall response (BOR) was comparable between the two groups.

OS outcomes for the chemotherapy group in patients with advanced PC with LM versus without LM are depicted in Figure 2A. Patients with LM had significantly shorter median OS (7.0 months, 95% CI: 6.0–8.8) compared to patients without LM (12.0 months, 95% CI: 8.7–18.0), with a log‐rank test *p* = 0.002. The unadjusted HR for death in patients with LM was 1.77 (95% CI: 1.22–2.58, *p* = 0.003). PFS outcomes for the chemotherapy group are shown in Figure 2B. Patients with LM also experienced significantly shorter PFS (5.0 months, 95% CI: 3.6–5.7) compared to those without LM (7.5 months, 95% CI: 5.4–9.0), with a log‐rank test *p* = 0.016 and an unadjusted HR of 1.58 (95% CI: 1.09–2.30, *p* = 0.017). Thus, LM was associated with significantly shorter OS in the chemotherapy regimen group.

In the combined ICI regimen group (CheckPAC, Triple‐R, and Influence studies), OS outcomes are depicted in Figure 2C. No significant differences in OS were observed between patients with LM (3.7 months, 95% CI: 3.0–4.4) and those without LM (5.0 months, 95% CI: 3.3–8.6), with a log‐rank test *p* = 0.21 and an unadjusted HR of 1.38 (95% CI: 0.83–2.27, *p* = 0.21). PFS outcomes for the ICI regimen group are shown in Figure 2D. Similarly, no significant differences were observed between patients with LM (1.7 months, 95% CI: 1.6–1.8) and those without LM (1.8 months, 95% CI: 1.6–3.9), with a log‐rank test *p* = 0.3 and an unadjusted HR of 1.34 (95% CI: 0.81–2.21, *p* = 0.3). Thus, LM was not significantly associated with OS or PFS in the ICI regimen group.

The LMR was only available for the Triple‐R and Influence study. One patient had missing data, leaving 44 patients for analysis. No significant differences were observed, although there was a trend towards higher LMR seen in patients without LM (Figure 3).

**Figure 3:**
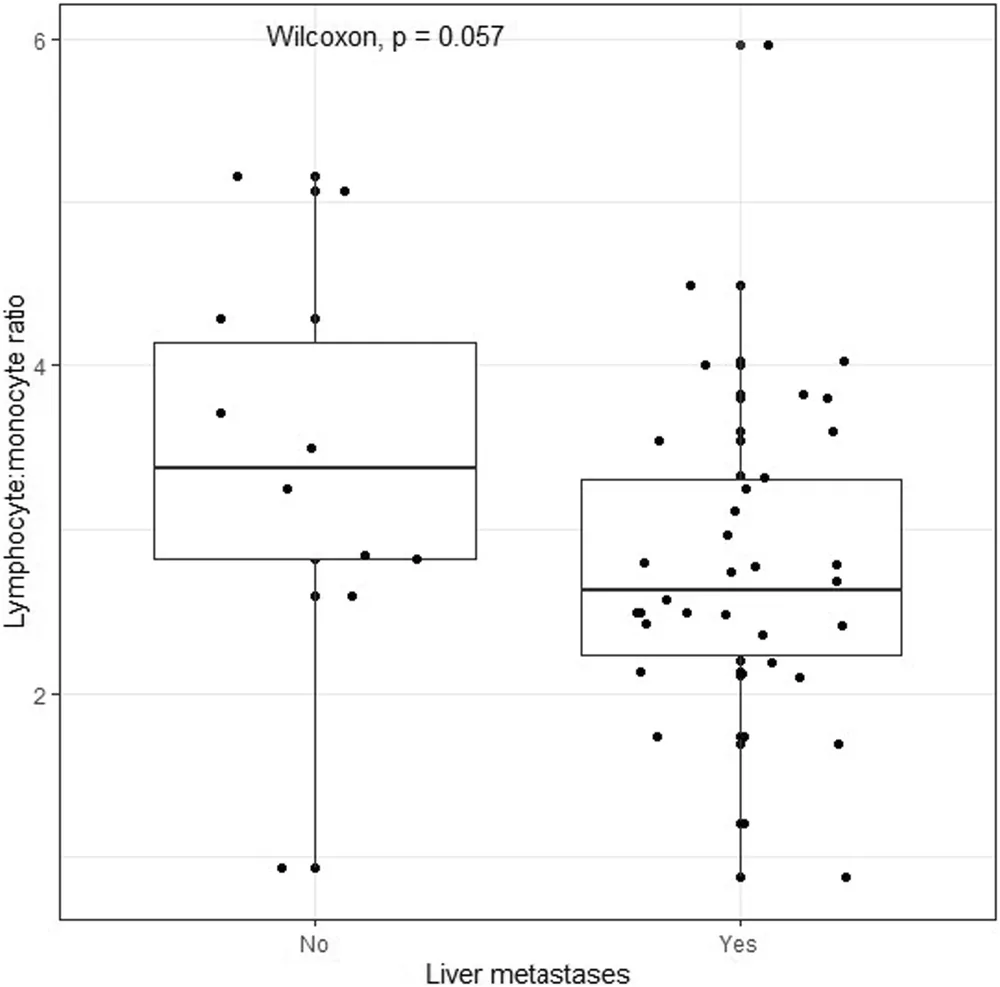
Lymphocyte‐to‐Monocyte Ratio (LMR) in the combined Triple‐R and Influence dataset (ICI regimen group), stratified by liver metastasis status. Liver metastasis status is coded as “No” for absence and “Yes” for presence. Each dot represents an individual patient. An arbitrary spread in the horizontal direction is added for clarity. Groups were compared using the Wilcoxon rank‐sum test (*p* = 0.057).

Multivariate analyses of OS are presented as forest plots in Figure 4A,B for the chemotherapy group and Figure 5A,B for the ICI regimen group. Age and sex did not significantly influence the effect of LM on OS. Similarly, no differences in survival were seen for NLR > 5 or ALC in either study. The study treatment arm with tocilizumab in chemotherapy group had a significant reduced HR in the multivariate analyses while high versus low YKL‐40 and IL‐6 had a significant increased HR (forest plots in Figure 4A,B) in the chemotherapy group, but not in the ICI regimen group (Figure 5A,B).

**Figure 4:**
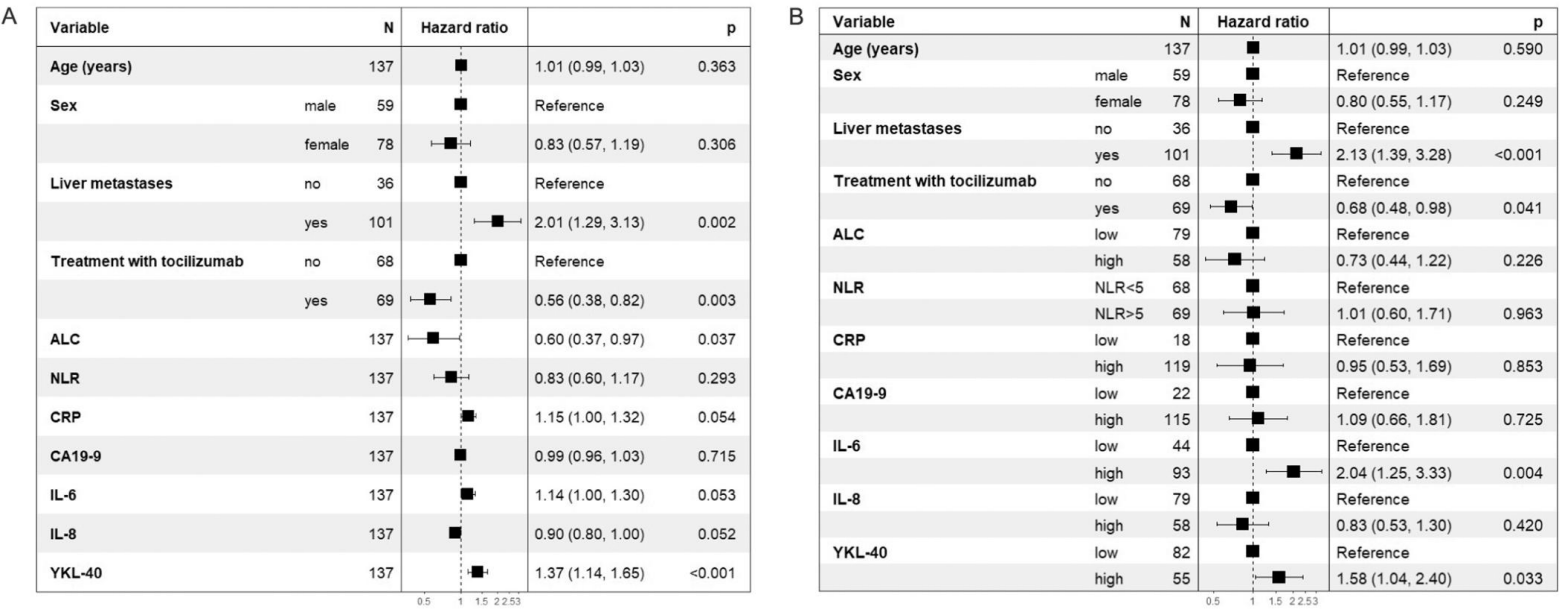
Forest plot of hazard ratios (HRs) for overall survival (OS) in the chemotherapy regimen group. Liver metastasis status is coded as “no” for absence and “yes” for presence. Tocilizumab treatment is coded as “no” for no receipt and “yes” for receipt. (A) ALC, NLR, CRP, CA 19‐9, IL‐6, IL‐8, and YKL‐40 were included as log2‐transformed continuous variables. (B) The same biomarkers were included as dichotomized variables, classified as high or low according to [≤ 37 and > 37 U/L for CA‐19‐9; ≤ 10 and > 10 mg/L for CRP; ≤ 5 and > 5 ng/L for IL‐6; ≤ 78 and > 78 ng/L for IL‐8; ≤ 200 and > 200 µg/L for YKL‐40; median cut‐off values for ALC was ≤ 5 and > 5 for NLR]. A total of 137 patients were included in the complete‐case analyses because of missing data for IL‐6, IL‐8, YKL‐40, and CRP. ALC, absolute lymphocyte count; CA 19‐9, carbohydrate antigen 19‐9; CRP, C‐reactive protein; NLR, neutrophil‐to‐lymphocyte ratio.

**Figure 5:**
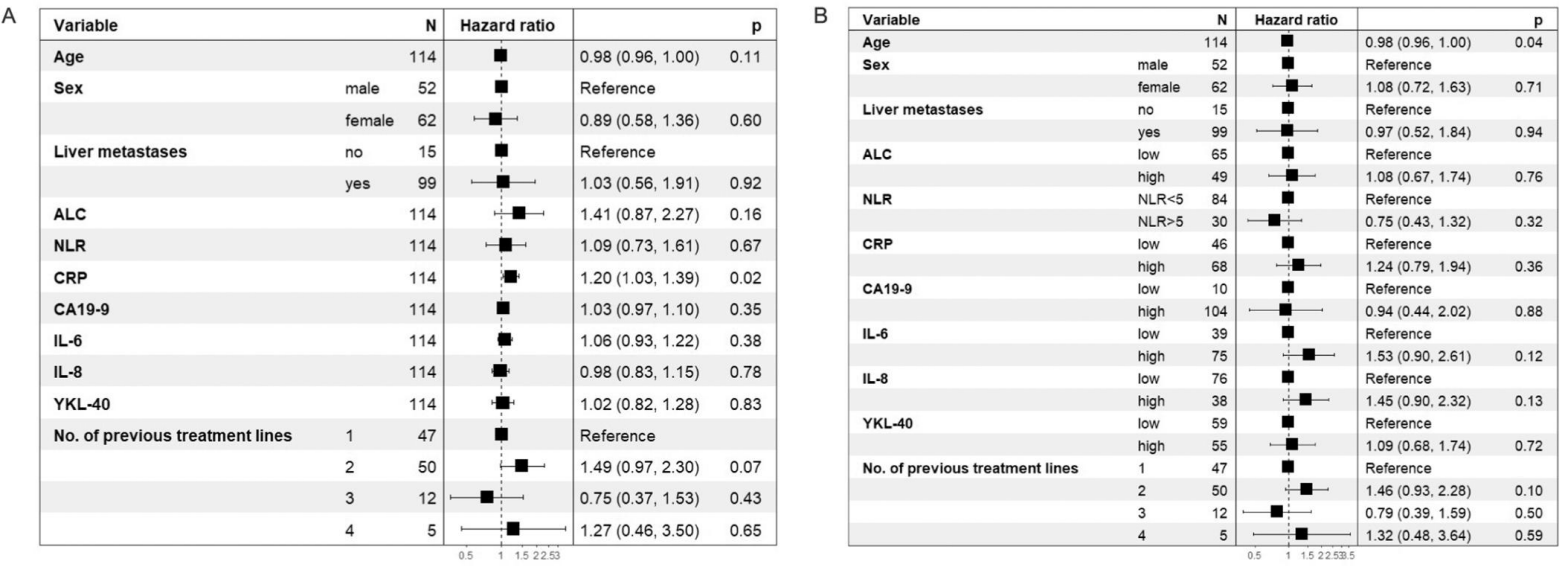
Forest plot of hazard ratios (HRs) for overall survival (OS) in the ICI‐regimen group. Liver metastasis status is coded as “no” for absence and “yes” for presence. (A) ALC, NLR, CRP, CA 19‐9, IL‐6, IL‐8, and YKL‐40 were included as log2‐transformed continuous variables. (B) The same biomarkers were included as dichotomized variables, classified as high or low according to [≤ 37 and > 37 U/L for CA‐19‐9; ≤ 10 and > 10 mg/L for CRP; ≤ 5 and > 5 ng/L for IL‐6; ≤ 78 and > 78 ng/L for IL‐8; ≤ 200 and > 200 µg/L for YKL‐40; median cut‐off values for ALC was ≤ 5 and > 5 for NLR]. A total of 114 patients were included in the complete‐case analyses because of missing data for IL‐6, IL‐8, YKL‐40, and CA 19‐9. ALC, absolute lymphocyte count; CA 19‐9, carbohydrate antigen 19‐9; CRP, C‐reactive protein; NLR, neutrophil‐to‐lymphocyte ratio.

In the chemotherapy group, some patients received the IL‐6 receptor inhibitor tocilizumab (Figure 5), and a small subset (*n* = 3) later transitioned to ICI regimens. A sensitivity analysis excluding these three patients showed no significant impact on OS results (*p* = 0.017, data not shown), though this remains a potential source of bias.

## 4 Discussion

In this post‐hoc study, we demonstrated that LM were associated with a significantly increased risk of death in patients with advanced PC receiving chemotherapy [22]. However, in patients treated with ICIs, LM did not significantly impact survival outcomes. These findings challenge the widely held assumption that LM universally impair ICI efficacy through immune‐suppressive mechanisms and raise important questions regarding whether differences in treatment, line of treatment (ICI studies were 2, 3. or later line of treatment), cohort size, or disease stage may have influenced our results.

### 4.1 LM and ICI Treatment: Biological Mechanisms and Possible Explanations

The expectation that LM would reduce ICI efficacy is based on extensive evidence from multiple cancer types, where the presence of LM is associated with poorer immunotherapy responses. In metastatic colorectal cancer without mismatch repair deficiency, LM have been linked to worse clinical outcomes with ICI treatment, including shorter OS, PFS, and lower disease control rates [8]. Similarly, findings from cancers with established ICI benefits, such as malignant melanoma, renal cell carcinoma, and lung cancer, suggest that LM may impair ICI efficacy, likely due to the immunosuppressive liver microenvironment [9]. In these malignancies, LM have been associated with significantly worse long‐term survival following nivolumab treatment [9]. However, in gastroesophageal cancer, a meta‐analysis of seven studies found no significant impact of LM on responses to ICI regimens [26]. These diverging findings highlight the complexity of the liver microenvironment across different malignancies and suggest that the impact of LM on ICI efficacy may be cancer‐type dependent. Our findings align more closely with studies in gastroesophageal cancer [26], as LM did not significantly affect survival outcomes in ICI‐treated PC patients. However, given the limited statistical power of our study, further research is needed to clarify these associations.

### 4.2 LM as a Prognostic Factor in Chemotherapy Treatment

Unlike the ICI group, we found that LM were a strong negative prognostic factor in patients receiving chemotherapy, consistent with prior studies indicating that LM contribute to systemic inflammation and disease progression in PC. Circulating immune biomarkers such as low ALC, high NLR, and elevated IL‐6 have been implicated in poor prognosis across multiple cancer types, including PC [27, 28, 29]. In agreement with a previous study from our group [19], although based on a different patient cohort, we found that high circulating levels of IL‐6 and YKL‐40 were associated with shorter OS. We observed no significant differences in ALC or NLR between patients with and without LM. This may reflect limitations in available data or heterogeneity within the patient population. A more detailed characterization of immune cell composition—using techniques such as flow cytometry or single‐cell RNA sequencing—could provide further insight into the immunological consequences of LM in PC.

### 4.3 Future Treatment Strategies: Targeting the LM TME in PC

Our findings highlight the negative prognostic impact of LM in PC and underscore the need for therapeutic strategies that address the immunosuppressive TME in LM. The liver plays a central role in systemic immune regulation, and while the PC TME have been associated with impaired cytotoxic T‐cell responses, expansion of myeloid‐derived suppressor cells (MDSCs), and macrophage‐driven immune evasion, the LM TME have been associated with unique neutrophil and macrophage polarization [30, 31, 32]. NLR in our study might not represent the subtypes of neutrophils associated with LM TME and immune escape. Monocyte levels were only available in the Triple‐R and Influence studies, limiting our ability to evaluate their prognostic role. However, a trend toward higher LMR was noted in patients with extrahepatic disease, suggesting potential differences in immune regulation based on metastatic pattern. This is consistent with emerging evidence that immunosuppressive macrophage subtypes drive LM‐mediated immune evasion in PC by reprogramming the TME via efferocytosis [32]. Preclinical data suggest that targeting macrophage efferocytosis can restore tumor immunity [32] and may enhance ICI efficacy.

Moreover, we have previously shown that an immune response against transforming growth factor beta (TGF‐β)—a master regulator of fibrosis and immunosuppression in PC—is associated with clinical benefit of ICI regimens [33]. This finding has led to an ongoing clinical trial investigating a TGF‐β peptide vaccine combined with ICIs and radiotherapy in patients with liver‐metastatic PC (ClinicalTrials.gov ID: NCT05721846).

PC is characterized by recurrent alterations in the oncogene Kirsten rat sarcoma viral oncogene homolog (KRAS), and tumor suppressor genes tumor protein (TP)53, cyclin‐dependent kinase inhibitor (CDKN2A), and SMA‐ and MAD‐related protein 4 (SMAD4) [7, 34]. These mutations may alter TGF‐β signaling at different levels and can be exploited therapeutically with potential targeted therapies to remodel the TME [34].

A multimodal approach targeting multiple facets of LM‐induced immune suppression—through efferocytosis inhibition, TGF‐β blockade, and potential targeted therapies for TME remodeling—may be key to overcoming resistance and improving outcomes in liver‐metastatic PC. Future research should prioritize clinical translation of these strategies to optimize treatment efficacy in this challenging patient population.

Ongoing clinical trials could reveal whether these multimodal strategies should be performed already at the time of surgery, with the promising results of personalized mRNA vaccines targeting neoantigens combined with immunotherapy (atezolizumab) and chemotherapy with 5‐flurouracil, leucovorin, oxaliplatin, and irinotecan (mFOLFIRINOX) in the adjuvant setting, with improved recurrence‐free survival and sustained long‐lived CD8+T cells [35, 36]. This has led to the phase II randomized clinical trial IMCODE003 (ClinicalTrials.gov ID: NCT05968326). These findings also highlight the urgent need for tailored treatment since PC is a heterogenic disease. However, to our knowledge, personalized vaccines in LM PC are currently not available and renders more research.

### 4.4 Limitations of the Study

This study is explanatory in nature, as the included trials were not explicitly designed to evaluate responses to chemotherapy or ICI regimens in PC patients with LM. The limited statistical power remains a concern, as most PC patients present with LM, resulting in a small comparator group without LM. Furthermore, the ICI regimens analyzed in these studies comprised ipilimumab and nivolumab, which may limit the generalizability of our findings to other ICIs.

While we observed a significant negative prognostic impact of LM in the chemotherapy‐treated cohort [22], the lack of a comparable ICI‐treated group in this dataset prevents us from drawing direct conclusions about LM‐mediated resistance to ICI therapy. Although prior studies in other malignancies suggest that LM can impair ICI efficacy [9], our data cannot establish causality between LM and differential ICI response in PC. Future studies should incorporate prospective stratification of PC patients based on metastatic pattern to determine whether LM‐driven immune suppression directly limits ICI efficacy.

Notably, recent studies suggest that PC patients with CD3+T‐cell‐enriched liver infiltration are less likely to develop LM [10], and that patients with extrahepatic metastatic PC may derive greater benefit from a combination of chemoradiation and ICI therapy [10]. Future studies should investigate whether baseline immune profiling can help refine treatment strategies for metastatic PC.

Additionally, our analyses may be influenced by the fact that some PC patients with LM received liver‐directed SBRT, potentially altering their sensitivity to ICIs and confounding the results. The inclusion of patients from different trials introduces variability in treatment regimens, which could further impact the interpretation of immunological differences.

Lastly, in the ICI group, approximately half of the patients had received at least 2 lines of prior treatment, and thus it would be interesting to explore an ICI cohort in first‐line to rule out biases.

## 5 Conclusions

Collectively, LM were associated with significantly shorter OS and PFS in patients with PC receiving first‐line chemotherapy. In contrast, LM did not significantly impact survival outcomes in patients with advanced, refractory PC treated with ICI‐based regimens. These findings suggest that while LM contribute to a poor prognosis in PC, their specific role in modulating ICI efficacy remains unclear. Prospective studies with larger cohorts are needed to validate these findings and to explore the potential of TME‐targeted approaches in patients with PC and LM.

## Author Contributions

**Laura K. Kjær:** conceptualization, methodology, writing – original draft, writing – review and editing. **Susann Theile:** conceptualization, methodology, writing – review and editing. **Kasper Madsen:** conceptualization, methodology, writing – review and editing. **Julia S. Johansen:** conceptualization, methodology, writing – review and editing. **Morten Orebo Holmström:** writing – review and editing. **Mads Hald Andersen:** writing – review and editing. **Inna M. Chen:** conceptualization, methodology, writing – original draft, writing – review and editing.

## Ethics Statement

The protocols, amendments, and informed consent forms were approved by the Ethics Committee of the Capital Region of Denmark before the start of the studies, that is, chemotherapy‐based study (ClinicalTrials.gov ID: NCT02767557), ICI‐based studies (NCT04258150, NCT02866383, and NCT05116917), and biomarker study BIOPAC (NCT03311776). Informed consent was obtained from each participant before inclusion in the studies.

## Consent

Informed consent for publication was obtained from each participant before inclusion in the studies.

## Conflicts of Interest

Inna M. Chen: Research Funding: Roche (Inst), Bristol Myers Squibb (Inst), Celgene (Inst), Genis (Inst), and Varian Medical Systems (Inst), AstraZeneca (Inst), and GENMAB (Inst). Travel, Accommodation Expenses: Roche, Bristol Myers Squibb, Celgene, Bayer, and AstraZeneca. Advisory Role: Amgen, AstraZeneca, Astella, ANOCCA, and Genmab. The remaining authors declare no conflicts of interest.

## Supporting information


Supporting File 1

Supporting File 2

## Data Availability

As the original trial protocols did not include a data‐sharing plan; thus, data from the trials will not be shared publicly, as data sharing was not included when ethical approvals were requested. Data‐sharing proposals can be directed to the corresponding author and will be reviewed and approved by the sponsor, investigator, and collaborators based on scientific merit. Subsequently approved, data can be shared through a secure online platform after signing a data‐access agreement.
