# Supplementary material for: Impact of Liver Metastases on Outcomes With Chemotherapy or Immune Checkpoint Inhibitors in Pancreatic Cancer
Source: Immun Inflamm Dis. 2026 Oct 6;14(10):e70545. doi: 10.1002/iid3.70545 (PMC13640521; doi:10.1002/iid3.70545)
Supplement: Supplementary file 1 [file IID3-14-e70545-s001.pdf]

Figure 1

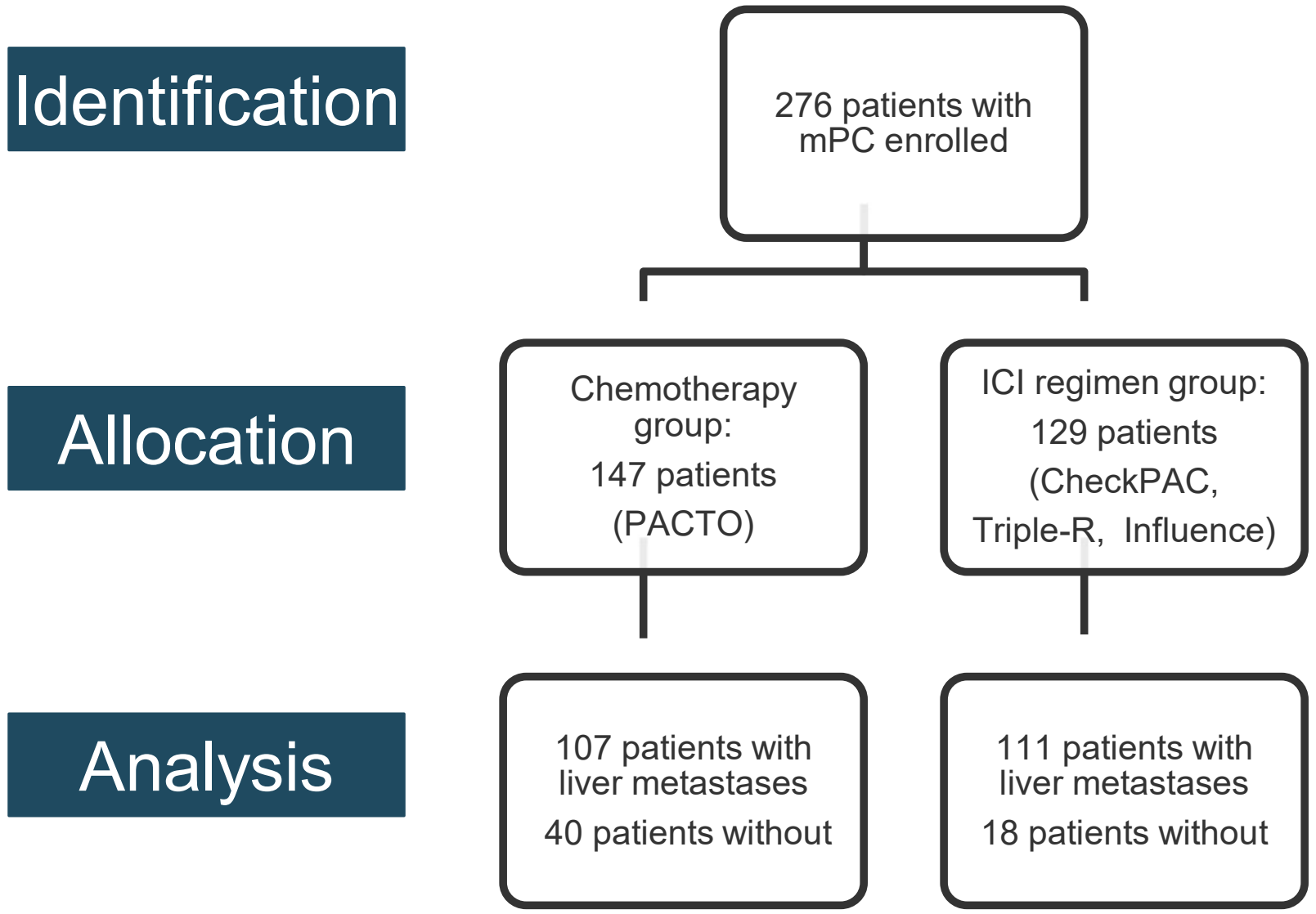
Identification
Allocation
Analysis
276 patients with mPC enrolled
Chemotherapy group:
147 patients
(PACTO)
ICI regimen group:
129 patients
(CheckPAC,
Triple-R, Influence)
107 patients with liver metastases
40 patients without
111 patients with liver metastases
18 patients without
