# Supplementary material for: Impact of Liver Metastases on Outcomes With Chemotherapy or Immune Checkpoint Inhibitors in Pancreatic Cancer
Source: Immun Inflamm Dis. 2026 Oct 6;14(10):e70545. doi: 10.1002/iid3.70545 (PMC13640521; doi:10.1002/iid3.70545)
Supplement: Supplementary file 2 [file IID3-14-e70545-s002.docx]

**IJC Manuscript Checklist**

Authors are required to comply with the policies listed below.

**Required for all article types.**

| I confirm that I have added to the title page of the manuscript the **institutional email addresses** of all corresponding authors. | ☒Yes |
| --- | --- |
| I confirm that I have added to the title page of the manuscript a statement about the paper being posted on a non-commercial **preprint server**, including the DOI and a link to the article. | ☐Yes  ☒Not Applicable |
| I confirm that all persons designated as authors qualify for authorship, and that all those who qualify for authorship – as noted in the authorship criteria in the [IJC Author Guidelines](https://onlinelibrary.wiley.com/page/journal/10970215/homepage/forauthors.html) are listed as authors. | ☒Yes |
| I confirm that I have not used any **Artificial Intelligence** Generated Content tools (e.g. ChatGPT) for the collection or interpretation of data, and for production of images or graphical elements of the manuscript. If I used such tools to create content or improve the readability and language of the paper, it is described, transparently and in detail in the Acknowledgement section. Further information is provided in the [IJC Author Guidelines](https://onlinelibrary.wiley.com/page/journal/10970215/homepage/forauthors.html). | ☒Yes |
| I confirm that the work reported in the paper has been performed by the authors, unless clearly specified in the text. | ☒Yes |

**Required for Research Article, Short Reports and Special Reports**

| I confirm that I have included at the end of the manuscript text, before the references, an ”**Ethics statement**” about ethical approval of the study and, if required, informed consent. | ☒Yes  ☐Not Applicable |
| --- | --- |
| I confirm that this **clinical trial** has been prospectively registered in a public trial registry, and I have included in the “Ethics statement” the name of the registry and the ***registration number***. | ☒Yes  ☐Not Applicable |
| I confirm that my article contains a [***Data Availability Statement***](https://authorservices.wiley.com/author-resources/Journal-Authors/open-access/data-sharing-citation/data-sharing-policy.html#standardtemplates). | ☒Yes |
| I confirm that I have included ***a citation*** (e.g., accession code, source code, URL) for available data in the manuscript. | ☒Yes  ☐Not Applicable |
| I confirm that the **sequencing coverage and quality statistics** of the (epi)genomic next generation sequencing generated in this study are summarized in a [supplementary table](https://onlinelibrary.wiley.com/pb-assets/assets/10970215/Sequencing%20Coverage%20and%20Quality%20Statistics-1715863066147.pdf) according to the IJC guidelines. | ☐Yes  ☒Not Applicable |
| I confirm that the **authenticity of any human cell lines** used in this study has been proven by DNA profiling within the last three years and the required documentation has been provided as per our [policy](https://onlinelibrary.wiley.com/page/journal/10970215/homepage/forauthors.html#POLI). | ☐Yes  ☒No cell lines used |
| I confirm that I have added in the Material and Methods section of the main manuscript a ***statement*** certifying that all human cell lines have been **authenticated using STR** (or SNP) profiling within the last three years. All cell lines used are listed using the official cell line name and its Research Resource Identifier (RRID) as available in the ExPASy Cellosaurus database (e.g., HeLa (RRID:CVCL_0030)). In addition, the source/suppliers of all cell lines used has/have been provided. | ☐Yes  ☒No cell lines used |
| I confirm that all experiments were performed with **mycoplasma-free cells** and a respective *statement* was included in the Material and Methods section of the main manuscript. | ☐Yes  ☒Not Applicable |
